# Editorial: The liver’s dilemma: sensing real danger in a sea of PAMPs

**DOI:** 10.3389/fimmu.2025.1591911

**Published:** 2025-05-20

**Authors:** Ian N. Crispe, Andrea Henriques Pons

**Affiliations:** ^1^ Departments of Laboratory Medicine and Pathology, Surgery, and Immunology, University of Washington, Seattle, WA, United States; ^2^ Instituto Oswaldo CruzLaboratório de Inovações em Terapias, Ensino e Bioprodutos Fundação Oswaldo Cruz Instituto, Rio de Janiero, Brazil

**Keywords:** liver, tolerance, sinusoids, microcirculation, PAMP (pathogen-associated molecular pattern)

The Signal and the Noise is a monograph focused on why predictions are so often wrong. The central thesis is that the Signal, which may offer a valid basis for prediction, is buried is a much larger volume of irrelevant data, the Noise. The immune system has been dealing with the issue of the Signal and the Noise much longer than we have. In this Research Topic, we address this issue in the context of the liver.

The sinusoids of the liver sit at the confluence of systemic arterial blood from the hepatic artery, and portal venous blood returning from the intestine. This mixed blood flow contains products of digestion derived from food, natural toxins and chemicals of human manufacture, molecules synthesized by the intestinal microbiota including cell wall elements, and intact bacteria that may translocate the intestinal wall and gain access to the portal circulation. The liver, interposed between this flood of non-self-molecules and the systemic circulation with all the vital organs it serves, is an effective filter that depletes many of the potentially harmful molecules. Thus, diverse chemicals are conjugated to side-chains such as glucuronic acid which facilitates their excretion in the bile; intact bacteria are captured by Kupffer cells, liver-resident macrophages that are intercalated with the sinusoidal endothelial cells. Microbial molecules such as flagellin and Lipopolysaccharide endotoxin (LPS) expressing Pathogen-Associated Molecular Patters (PAMPs) encounter Toll-Like Receptors and an array of scavenger receptors that bind them and extract them from the circulation. The large cross-sectional area of the hepatic vascular bed renders the flow of blood slow and intermittent, and the extraction of such molecules highly efficient.

This presents a problem. In other tissues, engagement of PAMP receptors by their ligands results in the activation of the innate immune system, leading to acute inflammatory responses, and the potentiation of antigen-presenting cells, leading to T cell activation. When PAMPs indicate the presence of an infection, as they do in most tissues, these responses are appropriate and the tissue injury that results is usually a good trade-off for immune protection. But in the liver, the vast majority of the PAMPS originate from our non-pathogenic microbiota. They are the Noise, and if a potentially pathogenic microbe is present its Signal offers just a few notes, lost in a molecular cacophony. We therefore solicited contributions that address how the liver deals with PAMPs, in the hope that some light may be shed on this problem.

A novel hypothesis is proposed by Henriques-Pons et al. to account for the liver’s capacity to remain unresponsive to PAMPs in health, but initiate inflammation when the circulation of blood through the hepatic sinusoids is perturbed in disease. Specifically, they postulate that, like many other metabolic and immune functions of the liver, the capacity to respond to PAMPs is localized to specific sinusoidal segments where arterial perfusion predominates. In the presence of normal circulation patterns, portal venous blood enriched in PAMPs does not reach these segments, which remain highly responsive. However, the presence of any kind of liver damage will result in disruption of the normal patterns of blood flow, with exposure of sensitive zones of the liver vasculature to PAMPs, to which they make an innate immune response. Depending on the insult, this may result in useful host defense, as in acute viral hepatitis, or enhanced pathology.

Acute liver injury is difficult to induce in mice with sub-lethal doses of LPS alone, but the effect may be potentiated by D-Galactosamine, and Zhang et al. in this Research Topic used this model to identify a molecule circuit that promotes liver injury. They created mice that lack the Apoptosis Stimulating Protein of p53-2 (ASPP2), and these mice were protected from liver injury. The mechanism of protection was linked to autophagy and to increased IL-6, suggesting that autophagy may be one of the ways in which the adverse effects of innate immune stimulation are ameliorated.

An alternative mechanism of liver injury is explored by Ni et al., who review the known mechanisms of pyroptosis and the ways in which its occurrence in Kupffer cells may be activated by diverse caspases as well as Granzyme-B, leading to the cleavage of Gasdermin as a final common pathway. Pyroptosis with increased cell membrane permeability and K+ efflux is linked to activation of the NLRP3 inflammasome, which in turn releases a burst of pro-inflammatory cytokines that orchestrate the local accumulation of inflammatory cells. There is nothing to suggest that pyroptosis makes a distinction between pathogenic PAMPS and those associated with harmless members of the healthy microbiota, but in future it may be fruitful to investigate whether pyroptosis is selectively activated in the presence of dangerous, but not innocuous PAMPs.

Kremer et al. in this Research Topic address the ways in which Liver Sinusoidal Endothelial Cells (LSECs) may regulate T cell immune responses in the context of primary liver cancer. Here, the insult is inflammation caused by the growth of the cancer, so the innate immune stimuli are better thought of as Damage-Associated Molecular Patterns (DAMPs) rather than PAMPs. But the authors show that when cancer-associated signals are present over time, the LSECs express co-inhibitory molecules, and in three cell-type cultures they can dominantly suppress T cell activation even in the presence of DCs. A key nuance of CD8+ T cell activation under these conditions is that the CD8+ T cells continue to secrete IFN-γ but lose their cytotoxic function. This suggests that one way to modulate immunity in the presence of a sea of PAMPs is to allow immunity but modulate the effector functions to minimize tissue injury.

We cannot yet explain how the liver responds appropriately to real danger in a sea of PAMPs, but elements of the solution are starting to emerge. Some combination of micro-environmental compartmentalization, the pro- and anti-inflammatory effects of multiple liver cell types including Kupffer cells and LSECs, and the immune deviation of PAMP responses towards those that cause less tissue injury, may all gel into a comprehensive solution to this problem.

